# Gastroesophageal reflux disease, mood swings, and frozen shoulder: A two-sample, two-step Mendelian randomization study

**DOI:** 10.1097/MD.0000000000040301

**Published:** 2024-11-01

**Authors:** Qiu-Cheng Guo, He Cai, Wu Hong, Yin-Ying Chen, Qing Lu, Shu-Guang Zheng

**Affiliations:** a GuiZhou University of Traditional Chinese Medicine, GuiYang, China; b The First Affiliated Hospital of GuiZhou University of Traditional Chinese Medicine, GuiYang, China.

**Keywords:** frozen shoulder, gastroesophageal reflux disease, mood swings, MR-PRESSO, two-step Mendelian randomization

## Abstract

A Mendelian randomization (MR) study was undertaken to establish a causal link between gastroesophageal reflux disease (GERD) and frozen shoulder (FS), examining whether the risk of GERD with FS is mediated through mood fluctuations. Genetic loci from populations of independent European ancestry were selected as instrumental variables for GERD, FS, and mood swings. The primary analysis employed the inverse-variance weighted method supplemented by 3 additional analytical methods. This was conducted using two-sample and two-step MR analyses. This study explored the correlation and mediating effects of mood swings between GERD and FS. Our study employed heterogeneity and horizontal diversity, and sensitivity analysis was conducted using the leave-one-out method to explore the robustness of the results. In the two-sample MR analysis, for every 1-unit increase in the log-transformed odds ratio (OR) of GERD, the corresponding OR increased to 1.844 (inverse-variance weighting: OR = 1.844, 95% confidence interval: 1.47–2.30, *P* < .001). In the two-step MR analysis, we found that mood swings played a mediating role in the association between GERD and FS. We assessed this mediating effect using the delta method (b = 0.181, SE = 0.059, OR = 1.199, 95% confidence interval: 1.072–1.349). Analysis of the data using the above methods indicated that GERD is a risk factor for FS, and mood swings mediate between the 2. Therefore, GERD and mood swings should be included in the health management of patients with FS.

## 1. Introduction

Frozen shoulder (FS), also known as adhesive capsulitis, is among the most debated shoulder diseases, with ongoing disputes ranging from terminology to optimal treatment and prognosis.^[1]^ Based on Sophie Abrassart recommendation, the term “frozen shoulder” better describes the clinical presentation; thus, this study consistently used the term.^[2]^ Its characteristics include gradually worsening shoulder joint pain and decreased range of motion. This disease, affecting 2% to 5% of the general population, is caused by chronic inflammation of the tissues surrounding the joint capsule.^[3]^ FS negatively affects mental health, quality of life, and work efficiency.^[4]^ Suffering from FS leads to a prolonged incapacity to work. In Sweden, it results in costs of over 10 billion Swiss Francs and more than 100,000 days of sick leave per year.^[5]^ The underlying pathophysiology of FS is not yet fully understood genome-wide association studies (GWAS) can offer new insights into potential biological mechanisms and possible drug targets.^[6]^ Currently, the triggers or aggravating factors for FS are still unclear, and there are varying viewpoints on the risk factors for the shoulder joint from different perspectives.^[4,7,8]^ Understanding the epidemiology of FS is vital in this scenario. Recently, a study identified loci related to FS and confirmed type 1 diabetes as a risk factor for the joint conditions. The study also employed Mendelian randomization (MR) was used to test the causal relationship between diabetes, obesity, and periarthritis of the shoulder.^[9]^ However, the number of published GWAS studies on FS has been limited.

Gastroesophageal reflux disease (GERD) is a common ailment characterized by the backflow of stomach contents into the esophagus, causing symptoms, or complications. From 1990 to 2019, the total number of prevalent cases, incident cases, and years lived with disability increased by 77.53%, 74.79%, and 77.19%, respectively. This rising trend has severely impacted the health-related quality of life for patients, leading to recurrent symptoms and numerous bothersome complications.^[10]^ However, few studies have examined the link between GERD and FS, although research suggests a possible association. The presence of GERD increases the risk of shoulder stiffness after arthroscopic rotator cuff repair surgery.^[11]^ Retrospective studies have also found that GERD is one of the risk factors for FS.^[4]^ However, current research and reports on the relationship between GERD and FS are relatively scarce.

Recently, the connection between FS and emotional well-being has garnered increasing attention.^[12]^ Research has found a significant positive correlation between the cumulative incidence of FS and depression.^[13]^ In cases of FS accompanied by depression or anxiety, there is often increased pain, restricted function, and physical impairment.^[14,15]^ Additionally, mood swings are more readily observed in patients with GERD.^[16]^ Based on these findings, we chose to study the relationship among these 3 factors.

MR is a method that uses genetic variables as “instruments” to infer causal relationships between environmental exposures and disease outcomes.^[17]^ This analysis is based on 3 crucial assumptions. First, the genetic variation that serves as an instrument must be highly correlated with the exposure factor. Second, the selected genetic variation was not be associated with any confounding factors. Third, exposure is be the only pathway through which the genetic variation affects the outcome.^[18]^ MR has been widely used for causal inference, combining the concepts of random grouping and the random allocation of genetic variations to overcome many limitations of traditional observational epidemiological studies.^[19,20]^ Compared with traditional observational epidemiological studies, the main advantages of MR are that avoids confounding by environmental and behavioral factors, eliminates reverse causation, assesses lifetime cumulative exposure effects, and strengthens causal hypotheses to provide a basis for clinical trials. MR uses genetic variations for random grouping, overcoming many limitations of traditional observational research and more accurately inferring the causal relationship between environmental exposure and disease outcomes.^[10]^

To date, no MR analysis has specifically addressed the association between GERD and FS. Understanding the association between GERD and FS has significant implications for the research and prevention of these 2 conditions that severely affect the quality of life, aiding in the development of more effective health management strategies for patients with FS.

## 2. Information and methods

### 2.1. Data sources

#### 2.1.1. Genetic instrumental variables (IVs) for GERD

Summary data related to GERD and single nucleotide polymorphisms (SNPs) used in this study were obtained from the GWAS catalog (ebi.ac.uk).^[18]^ The data included 129,080 cases, 473,524 controls, and 2,320,781 single SNPs. We set a genome-wide significance threshold (*P* < 5 × 10^-8^) for GERD. Linkage disequilibrium was removed (kb > 10,000 and *R*^2^ < 0.001) to ensure close relevance between variables and exposure.^[21]^

#### 2.1.2. Genetic IVs for mood swings

The IVs for mood swings were obtained using data from the UKBiobank and processed through the phesant-derived variables from the GWAS pipeline by Ieu Open. This included 204,412 cases, 274,207 controls, and 9,854,867 SNPs.

#### 2.1.3. Genetic IVs for FC

The data for FS come from the FinnGen project, a unique study that combines genomic information with digital healthcare data from participants over 18 years of age residing in Finland. For more details, the official FinnGen website (https://www.finngen.fi/fi) can be accessed for more details. The dataset used in this study includes 2942 cases, 167,641 controls, and 16,380,317 SNPs.

### 2.2. Study design

This study obtained publicly available large-scale GWAS summary datasets through the IEU Open GWAS project (gwas.mrcieu.ac.uk).In the relevant original GWAS, all subjects signed informed consent and received ethical approval. Written informed consent was obtained from all the. Therefore, additional ethical approval was not required. This study employed bi-sample MR to investigate the causal relationship between GERD and FS. This study utilized 4 regression models were used to analyze the data: inverse-variance weighting (IVW), weighted median method, MR-Egger regression, and weighted mode method, with the statistical significance threshold set at *P* < .05. In the IVW method, summary statistics can be used to test for causal effects for correlated variables.^[22]^ The weighted median method can provide consistent estimates of causal effects even when some information comes from invalid IVs.^[23]^ MR-Egger regression can detect violations of standard IV assumptions and provide effect estimates that are not influenced by these violations.^[24]^ The weighted mode method shows smaller biases and lower type I error rates compared to other methods under the null hypothesis.^[25]^ Furthermore, an MR-Egger intercept was used to test for horizontal pleiotropy. MR-Egger can detect certain behaviors that violate the standard assumptions of the IVs. MR-Egger can be used to detect and correct biases caused by directional pleiotropy.^[26]^ Cochran Q statistics and funnel plots were used to examine the heterogeneity of SNPs.^[27]^ A leave-one-out sensitivity test was also employed to ensure the robustness and reliability of the MR results. The IVW method is prone to the influence of weak instrument bias. Opting for a weak IVs with an F-value >10 can effectively mitigate bias in IVW analysis.^[28]^ We also used PhenoScanner (PhenoScanner (cam.ac.uk)) to query SNPs and eliminated confounding factors.

In the first step of the two-step MR, the dual-sample MR method was used to calculate the causal effect of exposure on the mediator variable (β1). In the second step, the effect of the mediator on the outcome is calculated to obtain (β2). The 2 coefficients were then multiplied to obtain the mediation effect, when β0, β1, and β2 were all significant. This indicates that there is a causal relationship between the exposure and outcome. The mediator variable can partially mediate this relationship. As per the convention, β1*beta2 can be considered as the mediation effect between exposure and outcome. While β1 × β2/β0 indicates the ratio of the mediating effect of causality.^[29]^

### 2.3. Sensitivity analysis

Owing to the potential heterogeneity among IV, the estimation of causal effects may be biased. Our study employed the Cochran *Q* statistic of MR-Egger and the IVW method for heterogeneity testing to detect this heterogeneity. The results of the heterogeneity test are presented in the Appendix 1, Supplemental Digital Content, http://links.lww.com/MD/N873.

We utilized the intercept value returned by MR-Egger and its *P*-value to test the horizontal pleiotropy of the IV. If the intercept value is close to 0 (<0.1) and the *P*-value is >.05, we can exclude the horizontal pleiotropy effect of IV.^[30]^ To better validate potential outlier SNPs and horizontal pleiotropy, we also adopted the MR-Pleiotropy RESidual Sum and Outlier (MR-PRESSO) method. In this study, we did not observe any horizontal pleiotropic issues.

Data analysis was conducted using the TwoSampleMR package in the R 4.2.3 software through RStudio.

## 3. Results

In the two-sample MR analysis, for every unit increase in the log-transformed odds ratio (OR) of GERD, the corresponding OR increased to 1.844. (IVW method: OR = 1.844, 95% confidence interval (CI) 1.47–2.30, *P* < .001). In the two-step MR analysis, we discovered that mood swings played a mediating role in the association between GERD and FS. We evaluated this mediation effect using the delta method (b = 0.181, SE = 0.059, OR = 1.199 (1.072–1.349)). This means that mood swings explain 29.6% of the risk association between GERD and FS.

### 3.1. Two-sample MR estimates for GERD and FS

The MR analysis results for GERD and FS are (OR = 1.844, 95% CI 1.47–2.30, *P* < .05), with the F-statistic range for SNPs related to GERD being 29.7 to 96.0. This indicates a strong association between the IV and exposure (Table 1).

**Table 1:** MR estimation of the relationship between GERD and risk of FS.

Exposure	Outcome	Method	NSNP	OR (95% CI)	b	SE	*P*-value
GERD	FS	MR-Egger	65	1.457 (0.38–5.60)	0.3764322	0.6872177	5.86E-01
GERD	FS	Weighted median	65	1.846 (1.35–2.51)	0.6131422	0.158183	1.06E-04
GERD	FS	Inverse-variance weighted	65	1.844 (1.47–2.30)	0.6118476	0.1136556	7.31E-08
GERD	FS	Weighted mode	65	1.911 (0.96–3.78)	0.6463758	0.3486543	6.84E-02

CI = confidence interval, FS = frozen shoulder, GERD = gastroesophageal reflux disease, OR = odds ratio, SNPs = single nucleotide polymorphisms.

### 3.2. Two-sample MR estimates for GERD and mood swings

Results for GERD and mood swings (OR = 1.101, 95% CI 1.08–1.12, *P* < .01), between GERD and the risk of FS, via the mediating role of mood swings through relevant SNPs, a two-step MR study estimated the mediation (mood swings) effect on FS as (b2 = 1.881, SE = 0.589, *P* = .001) (Table 2). Among SNPs of mood swings, SNPs appearing in GERD were excluded, and the F-statistic range for the SNPs used for mood swings is 29.78 to 63.74, indicating that weak instrument bias is unlikely to affect SNP selection.

**Table 2:** MR estimation of the relationship between GERD and risk of mood swings.

Exposure	Outcome	Method	NSNP	OR (95% CI)	b	SE	*P*-value
GERD	Mood swings	MR-Egger	77	1.137 (1.03–1.25)	0.12822535	0.047923617	9.46E-03
GERD	Mood swings	Weighted median	77	1.085 (1.06–1.10)	0.08142408	0.008001115	2.52E-24
GERD	Mood swings	Inverse-variance weighted	77	1.101 (1.08–1.12)	0.09645988	0.007539588	1.78E-37
GERD	Mood swings	Weighted mode	77	1.080 (1.04–1.12)	0.07718296	0.020133171	2.88E-04

CI = confidence interval, OR = odds ratio, SNPs = single nucleotide polymorphisms.

### 3.3. Two-step MR analysis

The MR analysis of GERD, mood swings, and FS showed that β0, β1, and β2 were significant. This suggests that mood swings mediate the association between GERD and FS, leading to an increased risk of FS. Specifically, the mediation effect is β1 × β2 = 0.181, and the direct effect is β0 − (β1 × β2), with a β0 result being of 0.430.

We utilized the delta method to evaluate the numerical value of the mediation effect (b = 0.181, SE = 0.059, OR = 1.199 (1.072–1.349)), validating the existence and credibility of this mediation effect. As OR > 1, it indicates that this mediation effect is a risk factor for FS. The proportion of mediation effect was 29.6%. This research provides an important foundation for a deeper understanding of the interrelationships between GERD, mood swings, and FS.

**Figure 1.**
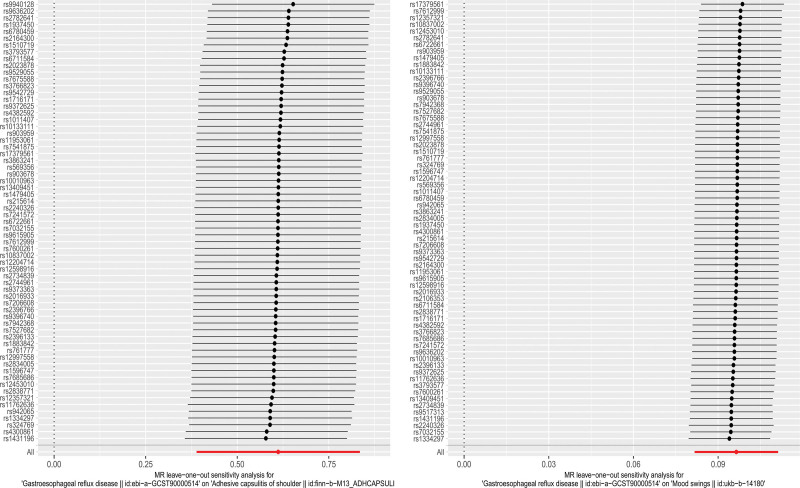
Leave-one-out plot of the Mendelian randomization analysis. (Left: GERD–FS. Right: GERD–mood swings). FS = frozen shoulder, GERD = gastroesophageal reflux disease.

The leave-one-out sensitivity analysis showed that regardless of which GERD was eliminated, the results related to mood swings’ SNPs did not change significantly, indicating a high robustness of the MR findings in this study (Fig. 1).

### 3.4. Sensitivity analysis

**Figure 2.**
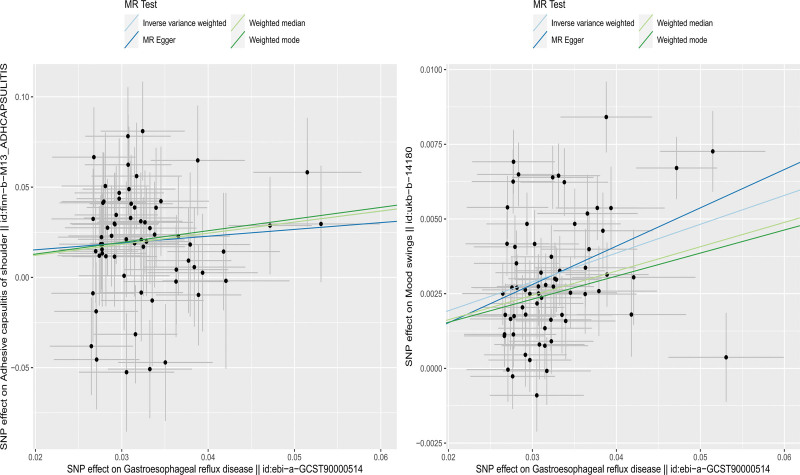
Scatter plot of the Mendelian randomization analysis. (Left: GERD–FS. Right: GERD–mood swings). FS = frozen shoulder, GERD = gastroesophageal reflux disease.

**Figure 3.**
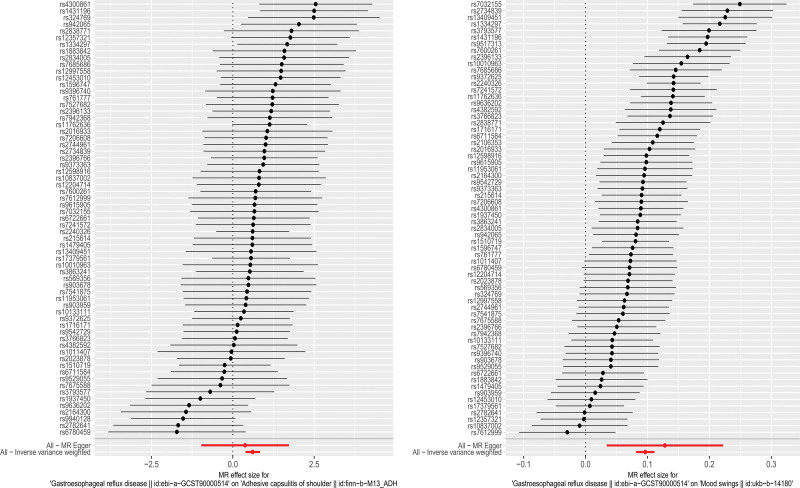
Forest plot of the Mendelian randomization analysis. (Left: GERD–FS. Right: GERD–mood swings). FS = frozen shoulder, GERD = gastroesophageal reflux disease.

**Figure 4.**
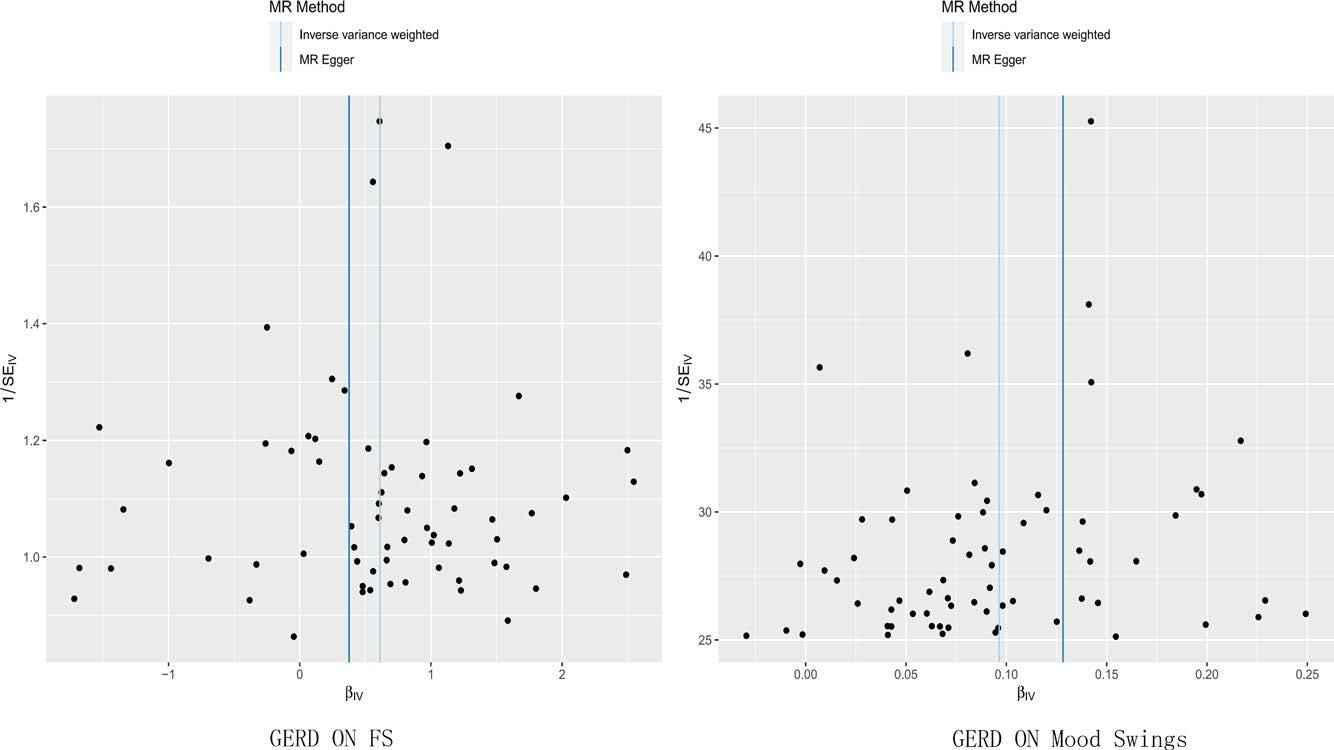
Funnel plot of the Mendelian randomization analysis. (Left: GERD–FS. Right: GERD–mood swings). FS = frozen shoulder, GERD = gastroesophageal reflux disease.

The MR-Pleiotropy RESidual Sum and Outlier (MR-PRESSO) method did not detect potential pleiotropy in the MR estimation in this study. Figures 2 to 4, display the scatter plots, forest plot, and funnel plot.

FS, in recent studies, is still considered an enigmatic disease, with current treatment approaches yielding limited results. The challenge in successfully treating this ailment lies in the insufficient understanding of its risk factors and the underlying pathophysiology. The aim of this study was to provide some support for understanding the causal relationships and mechanisms affecting up to 5% of the global population with FS.^[31]^

GERD, as a prevalent condition, is one of the 10 most common diseases in males aged > 50 years of age.^[32]^ Over the past 29 years, the global prevalence of GERD has increased by 77.53%.^[10]^ Both this disease and FS exhibit a higher proportion of female cases in term of prevalence and incidence, potentially indicating a connection.^[33]^ Recently, the association between GERD and FS has gained recognition. This study investigates the relationship between FS and GERD from a genetic perspective, and to some extent, explains the mechanisms underlying their association.

The mucosa of patients with GERD patients produces a higher amount of various cytokines (such as interleukin-1 [IL-1] IL-1β, tumor necrosis factor-α, and IL-6) and chemokines (such as Intercellular Adhesion Molecule 1). These inflammatory mediators activate immune cell recruitment and migration, which may be involved in the pathophysiology of FS.^[34]^ Moreover, inflammatory cytokines are crucial in disease progression, especially IL-1 are crucial for disease progression. IL-1 plays a role in cell proliferation, immune cell recruitment, and smooth muscle cell contraction.^[35]^ The expression levels of IL-1β and tumor necrosis factor-α in the synovial tissue of FS patients are higher than those in normal synovial tissue.^[36]^ These different cytokine induction pathways lead to TGF-β stimulation, resulting in overexpression of fibrotic factors in capsules or synovial/capsular fibroblasts.^[37]^ Concurrently, Intercellular Adhesion Molecule 1 induces intercellular adhesion, thereby facilitating intercellular communication, T-cell mediated defense mechanisms, and inflammatory responses.^[38]^ Symptoms caused by GERD such as gastric burning and difficulty swallowing, can lead to mood swings to a certain extent.^[39]^ Observations have found that patients with GERD are more prone to mood swings.^[40]^ Meanwhile, clinical studies have indicated that anxiety is a risk factor for the onset of FS,^[12]^ and mood swings can affect the treatment outcomes of FS.^[41]^ Therefore, this study focuses on the connections among the 3.

Moreover, females are more prone to emotional fluctuations than males.^[42]^ This might be a reason for the higher incidence rate of FS among females. In the population with FS, individuals with psychological disorders report more severe shoulder pain and functional limitation.^[15]^ Psychological factors (such as emotional distress) might affect the intensity of pain and the persistence of disability.^[43]^ Research has discovered that in individuals experiencing mood swings, the levels of tumor necrosis factor and pro-inflammatory cytokine IL-6 are elevated.^[44]^ To some extent, these studies revealed the pathophysiological connection between GERD, mood swings, and FS. In this study, we confirmed the causal effect between GERD and FS, and found that mood swings act as a mediator in the causal effect of GERD on FS. Although the mutual connections among the 3 has been noted, this study is the first to link them together.

Our two-sample, two-step MR study has certain advantages, In MR studies, the lifelong exposure to alleles of subjects helps to reduce the interference of other potential factors on the research results. Additionally, we used SNPs with a high association strength (F > 29) as IVs. Hence, our MR study can effectively avoided the common issues of confounding bias and reverse causality observed in observational studies. However, our study also has certain limitations. Firstly, the subjects of this study were of European descent, therefore, caution is needed when generalizing the results to other populations. Secondly, the data we used came from public databases, which limits our ability to conduct a segmented analysis of factors such as gender. Finally, although our study can provide preliminary insights into the relationship between GERD, mood swings, and FS, the complex mechanisms connecting the 3 still require further in-depth exploration.

## 4. Conclusion

In summary, this study provides support through MR analysis, confirming the causal relationship between GERD and FS. GERD increases the risk of FS and reveals the mediating role of mood swings. Therefore, GERD and mood swings should be included in the health management of patients with FS.

## Author contributions

**Data curation:** Qiu-Cheng Guo.

**Methodology:** Qiu-Cheng Guo, Yin-Ying Chen, Qing Lu.

**Resources:** Qiu-Cheng Guo.

**Validation:** He Cai.

**Visualization:** Qiu-Cheng Guo, Wu Hong.

**Writing – original draft:** Qiu-Cheng Guo.

**Writing – review & editing:** Shu-Guang Zheng.

## Supplementary Material



## References

[1] Pandey V Madi S. Clinical guidelines in the management of frozen shoulder: an update! Indian J Orthop. 2021;55:299–309. 33912325 10.1007/s43465-021-00351-3 PMC8046676

[2] Abrassart S Kolo F Piotton S. ‘Frozen shoulder’ is ill-defined. How can it be described better? EFORT Open Rev. 2020;5:273–9. 32509332 10.1302/2058-5241.5.190032 PMC7265085

[3] Neviaser AS Neviaser RJ. Adhesive capsulitis of the shoulder. J Am Acad Orthop Surg. 2011;19:536–42. 21885699 10.5435/00124635-201109000-00004

[4] Jacob L Gyasi RM Koyanagi A Haro JM Smith L Kostev K. Prevalence of and risk factors for adhesive capsulitis of the shoulder in older adults from Germany. J Clin Med. 2023;12:669. 36675599 10.3390/jcm12020669 PMC9866675

[5] Bouaicha S Wieser K Kriechling P Scholz-Odermatt SM. A large-scale assessment of the healthcare burden of adhesive capsulitis of the shoulder joint. Swiss Med Wkly. 2020;150:w20188. 32083705 10.4414/smw.2020.20188

[6] Gordon JA Farooqi AS Rabut E. Evaluating whole-genome expression differences in idiopathic and diabetic adhesive capsulitis. J Shoulder Elbow Surg. 2022;31:e1–e13. 34352401 10.1016/j.jse.2021.06.016 PMC8665043

[7] Kraal T Lübbers J van den Bekerom MPJ. The puzzling pathophysiology of frozen shoulders - a scoping review. J Exp Orthop. 2020;7:91. 33205235 10.1186/s40634-020-00307-w PMC7672132

[8] Tie K Wang H Yang X Ni Q Chen L. Analysis of risk factors for advanced age in patients with frozen shoulder. Aging Clin Exp Res. 2023;35:615–20. 36723857 10.1007/s40520-023-02347-5

[9] Green HD Jones A Evans JP. A genome-wide association study identifies 5 loci associated with frozen shoulder and implicates diabetes as a causal risk factor. PLoS Genet. 2021;17:e1009577. 34111113 10.1371/journal.pgen.1009577 PMC8191964

[10] Zhang D Liu S Li Z Wang R. Global, regional and national burden of gastroesophageal reflux disease, 1990-2019: update from the GBD 2019 study. Ann Med. 2022;54:1372–84. 35579516 10.1080/07853890.2022.2074535 PMC9122392

[11] Cucchi D Menon A Feroldi FM Boerci L Randelli PS. The presence of gastroesophageal reflux disease increases the risk of developing postoperative shoulder stiffness after arthroscopic rotator cuff repair. J Shoulder Elbow Surg. 2020;29:2505–13. 32711105 10.1016/j.jse.2020.07.002

[12] Aïm F Chevallier R Marion B Klouche S Bastard C Bauer T. Psychological risk factors for the occurrence of frozen shoulder after rotator cuff repair. Orthop Traumatol Surg Res. 2022;108:103212. 35077897 10.1016/j.otsr.2022.103212

[13] Jacob L Koyanagi A Oh H. Association between adhesive capsulitis and depression: a five-year retrospective cohort study including 58,516 adults from Germany. J Psychiatr Res. 2022;155:395–400. 36182769 10.1016/j.jpsychires.2022.09.040

[14] Ebrahimzadeh MH Moradi A Bidgoli HF Zarei B. The relationship between depression or anxiety symptoms and objective and subjective symptoms of patients with frozen shoulder. Int J Prev Med. 2019;10:38. 30967924 10.4103/ijpvm.IJPVM_212_17 PMC6425770

[15] Ding H Tang Y Xue Y. A report on the prevalence of depression and anxiety in patients with frozen shoulder and their relations to disease status. Psychol Health Med. 2014;19:730–7. 24382210 10.1080/13548506.2013.873814

[16] Bai P Bano S Kumar S. Gastroesophageal reflux disease in the young population and its correlation with anxiety and depression. Cureus. 2021;13:e15289. 34194886 10.7759/cureus.15289 PMC8236209

[17] Burgess S Davey Smith G Davies NM. Guidelines for performing Mendelian randomization investigations: update for summer 2023. Wellcome Open Res. 2019;4:186. 32760811 10.12688/wellcomeopenres.15555.1 PMC7384151

[18] Lawlor DA Harbord RM Sterne JA Timpson N Davey Smith G. Mendelian randomization: using genes as instruments for making causal inferences in epidemiology. Stat Med. 2008;27:1133–63. 17886233 10.1002/sim.3034

[19] Skrivankova VW Richmond RC Woolf BAR. Strengthening the reporting of observational studies in epidemiology using mendelian randomization: the STROBE-MR statement. JAMA. 2021;326:1614–21. 34698778 10.1001/jama.2021.18236

[20] Tin A Köttgen A. Mendelian randomization analysis as a tool to gain insights into causes of diseases: a primer. J Am Soc Nephrol. 2021;32:2400–7. 34135084 10.1681/ASN.2020121760 PMC8722812

[21] Davies NM Holmes MV Davey Smith G. Reading Mendelian randomisation studies: a guide, glossary, and checklist for clinicians. BMJ. 2018;362:k601. 30002074 10.1136/bmj.k601 PMC6041728

[22] Zheng J Baird D Borges MC. Recent developments in mendelian randomization studies. Curr Epidemiol Rep. 2017;4:330–45. 29226067 10.1007/s40471-017-0128-6 PMC5711966

[23] König IR Greco FMD. Mendelian randomization: progressing towards understanding causality. Ann Neurol. 2018;84:176–7. 30014502 10.1002/ana.25293 PMC6221001

[24] Burgess S Dudbridge F Thompson SG. Combining information on multiple instrumental variables in Mendelian randomization: comparison of allele score and summarized data methods. Stat Med. 2016;35:1880–906. 26661904 10.1002/sim.6835 PMC4832315

[25] Bowden J Davey Smith G Haycock PC Burgess S. Consistent estimation in Mendelian randomization with some invalid instruments using a weighted median estimator. Genet Epidemiol. 2016;40:304–14. 27061298 10.1002/gepi.21965 PMC4849733

[26] Bowden J Davey Smith G Burgess S. Mendelian randomization with invalid instruments: effect estimation and bias detection through Egger regression. Int J Epidemiol. 2015;44:512–25. 26050253 10.1093/ije/dyv080 PMC4469799

[27] Hartwig FP Davey Smith G Bowden J. Robust inference in summary data Mendelian randomization via the zero modal pleiotropy assumption. Int J Epidemiol. 2017;46:1985–98. 29040600 10.1093/ije/dyx102 PMC5837715

[28] Lin Z Deng Y Pan W. Combining the strengths of inverse-variance weighting and Egger regression in Mendelian randomization using a mixture of regressions model. PLoS Genet. 2021;17:e1009922. 34793444 10.1371/journal.pgen.1009922 PMC8639093

[29] Cui Y Lu W Shao T Zhuo Z Wang Y Zhang W. Severe mental illness and the risk of breast cancer: a two-sample, two-step multivariable Mendelian randomization study. PLoS One. 2023;18:e0291006. 37656762 10.1371/journal.pone.0291006 PMC10473543

[30] Verbanck M Chen CY Neale B Do R. Detection of widespread horizontal pleiotropy in causal relationships inferred from Mendelian randomization between complex traits and diseases. Nat Genet. 2018;50:693–8. 29686387 10.1038/s41588-018-0099-7 PMC6083837

[31] de la Serna D Navarro-Ledesma S Alayón F López E Pruimboom L. A comprehensive view of frozen shoulder: a mystery syndrome. Front Med (Lausanne). 2021;8:663703. 34046418 10.3389/fmed.2021.663703 PMC8144309

[32] Fenter TC Naslund MJ Shah MB Eaddy MT Black L. The cost of treating the 10 most prevalent diseases in men 50 years of age or older. Am J Manag Care. 2006;12(4 Suppl):S90–8. 16551207

[33] Pandey V Chidambaram R Modi A. Trends in practice among shoulder specialists in the management of frozen shoulder: a consensus survey. Orthop J Sports Med. 2022;10:23259671221118834. 36250030 10.1177/23259671221118834 PMC9561673

[34] Altomare A Guarino MP Cocca S Emerenziani S Cicala M. Gastroesophageal reflux disease: update on inflammation and symptom perception. World J Gastroenterol. 2013;19:6523–8. 24151376 10.3748/wjg.v19.i39.6523 PMC3801363

[35] Kim YS Kim JM Lee YG Hong OK Kwon HS Ji JH. Intercellular adhesion molecule-1 (ICAM-1, CD54) is increased in adhesive capsulitis. J Bone Joint Surg Am. 2013;95:e181–8. 10.2106/JBJS.K.00525 23426775

[36] Lho YM Ha E Cho CH. Inflammatory cytokines are overexpressed in the subacromial bursa of frozen shoulder. J Shoulder Elbow Surg. 2013;22:666–72. 22999851 10.1016/j.jse.2012.06.014

[37] Alghamdi A Alyami AH Althaqafi RMM. Cytokines’ role in the pathogenesis and their targeting for the prevention of frozen shoulder: a narrative review. Cureus. 2023;15:e36070. 37056530 10.7759/cureus.36070 PMC10092900

[38] Singh M Thakur M Mishra M. Gene regulation of intracellular adhesion molecule-1 (ICAM-1): a molecule with multiple functions. Immunol Lett. 2021;240:123–36. 34715236 10.1016/j.imlet.2021.10.007

[39] Kahrilas PJ. Clinical practice. Gastroesophageal reflux disease. N Engl J Med. 2008;359:1700–7. 18923172 10.1056/NEJMcp0804684 PMC3058591

[40] Quach DT Phan BT. A long duration of reflux symptoms is the predominant risk factor for depression in Vietnamese patients with gastroesophageal reflux disease. Neuropsychiatr Dis Treat. 2022;18:2141–50. 36176921 10.2147/NDT.S381892 PMC9514266

[41] Debeer P Commeyne O De Cupere I. The outcome of hydrodilation in frozen shoulder patients and the relationship with kinesiophobia, depression, and anxiety. J Exp Orthop. 2021;8:85. 34591188 10.1186/s40634-021-00394-3 PMC8484410

[42] Chui H Hoppmann CA Gerstorf D Walker R Luszcz MA. Social partners and momentary affect in the oldest-old: the presence of others benefits affect depending on who we are and who we are with. Dev Psychol. 2014;50:728–40. 23895170 10.1037/a0033896 PMC4854631

[43] Martinez-Calderon J Meeus M Struyf F Miguel Morales-Asencio J Gijon-Nogueron G Luque-Suarez A. The role of psychological factors in the perpetuation of pain intensity and disability in people with chronic shoulder pain: a systematic review. BMJ Open. 2018;8:e020703. 10.1136/bmjopen-2017-020703 PMC5905738 29654040

[44] Bavaresco DV da Rosa MI Uggioni MLR. Increased inflammatory biomarkers and changes in biological rhythms in bipolar disorder: a case-control study. J Affect Disord. 2020;271:115–22. 32479306 10.1016/j.jad.2020.03.073

