# Supplementary material for: Gastroesophageal reflux disease, mood swings, and frozen shoulder: A two-sample, two-step Mendelian randomization study
Source: Medicine (Baltimore). 2024 Nov 1;103(44):e40301. doi: 10.1097/MD.0000000000040301 (PMC11537667; doi:10.1097/MD.0000000000040301)
Supplement: Supplementary file 1 [file medi-103-e40301-s001.docx]

# SUpplemental Date Set

| Analysis of Directional Horizontal Pleiotropy | | | | | | | | | | | |
| --- | --- | --- | --- | --- | --- | --- | --- | --- | --- | --- | --- |
| Exposure | | Outcome | | Egger_intercept | | se | | | | pval | |
| Gastroesophageal reflux disease | | Adhesive capsulitis of shoulder | | 0.007758731 | | 0.02233278 | | | | 0.7294379 | |
| Gastroesophageal reflux disease | | Mood swings | | -0.001038883 | | 0.001547646 | | | | 0.5044665 | |
| Mood swings | | Adhesive capsulitis of shoulder | | -0.03319215 | | 0.02641907 | | | | 0.2144912 | |
| Heterogeneity Statistics | | | | | | | | | |  |  |
| exposure | | outcome | | method | | Q | Q_df | Q_pval | |  |  |
| Gastroesophageal reflux disease | | Adhesive capsulitis of shoulder | | MR Egger | | 65.92666 | 63 | 3.76E-01 | |  |  |
| Gastroesophageal reflux disease | | Adhesive capsulitis of shoulder | | Inverse variance weighted | | 66.05297 | 64 | 4.06E-01 | |  |  |
| Gastroesophageal reflux disease | | Mood swings | | MR Egger | | 196.8674 | 64 | 1.93E-15 | |  |  |
| Gastroesophageal reflux disease | | Mood swings | | Inverse variance weighted | | 198.2535 | 65 | 2.12E-15 | |  |  |
| Mood swings | | Adhesive capsulitis of shoulder | | MR Egger | | 56.30954 | 53 | 0.3521679 | |  |  |
| Mood swings | | Adhesive capsulitis of shoulder | | Inverse variance weighted | | 57.98657 | 54 | 0.3305567 | |  |  |
